# Supplementary material for: INO10, a Chaga Mushroom Extract, Alleviates Alzheimer’s Disease-Related Pathology and Cognitive Deficits in 3xTg-AD Mice
Source: Int J Mol Sci. 2025 May 15;26(10):4729. doi: 10.3390/ijms26104729 (PMC12111798; doi:10.3390/ijms26104729)
Supplement: Supplementary file 1 [file ijms-26-04729-s001.zip › ijms-3574664-supplementary.pdf]

Supplementary information for

INO10, a chaga mushroom extract, alleviates Alzheimer's disease-related pathology and cognitive deficits in 3xTg-AD mice.

So Young Ban[a,b], Thi Thuong Do[b], Jang-Won Pyo[b], Minho Moon[c,d,*], Jong-Tae Park[a,b,*]

[a]Department of Food Science and Technology, Chungnam National University, Daejeon 34134, Republic of Korea

[b]CARBOEXPERT Inc., Daejeon 34134, Republic of Korea

[c]Department of Biochemistry, College of Medicine, Konyang University, Daejeon 35365, Republic of Korea

[d]Research Institute for Dementia Science, Konyang University, Daejeon 35365, Republic of Korea

[*]Corresponding authors.

E-mail address: hominmoon@konyang.ac.kr (Minho Moon), jtpark@cnu.ac.kr (Jong-Tae Park)

Supplementary information

Table S1. Purity and composition analysis of INO10/γ-CD

The purity and composition of INO10/γ-CD were analyzed at the Institute of Agricultural Science, Chungnam National University (Daejeon, Republic of Korea). The results indicated that the crud protein content was 0.62% and the crude fat content was 0.25%, both of which were below 1% (Test Report No. 2501545).

Content	Result (%)	
Crude protein	0.62	
Crude fat	0.25	

Table S2. The mobile phase gradients and HPLC-ELSD instrument conditions.

The mobile phase gradients			HPLC-ELSD condition				
Time (min)	A (%) DW	B (%) Methanol	Injection volume (mL)	Flow rate (mL/min)	Gas flow (L/min)	Drift-tube temperature (°C)	Column temperature (°C)
0	20	80	10	1	3	90	50
5.0	5	95					
10.0	5	95					
10.1	0	100					
20.0	0	100					
20.1	20	80					
25	20	80					

Table S3. The mobile phase gradients and HPLC-MS/MS instrument condition.

The mobile phase gradients			HPLC-MS/MS condition			
Time (min)	A (%) DW	B(%) Methanol	Injection volume (mL)	Flow rate (mL/min)	Column temperatu re (°C)	Mode
0	25	75	2	0.2	40	Positive
2	25	75				
6	10	90				
8	3	97				
16	0	100				
17	25	75				
20	25	75				

Table S4. The positive mode and MS/MS conditions.

Positive mode conditions						
Dry gas temperature (°C)	Dry gas flow rate (L/min)	Nebulizer pressure (psi)	Sheath gas temperature (°C)	Sheath gas flow rate (L/min)	Capillary voltage (V)	Nozzle voltage (V)
270	10	40	300	11	3500	500

Table S5. Genes and PCR primers used in this study.

Gene	Forward	Reverse
GAPDH	ACCCAGAAGACTGTGGATGG	CCTGTTGCTGTAGCCGTATTC
IL-1β	AGAAGCTGTGGCAGCTACCTG	GCCGTCTTTCATTACACAGG
IL-6	AGCCAGAGTCCTTCAGAGA	GTCAGATACCTGACAACAGG
TNF-α	TCTTCTCATTCCTGCTTGTGG	GGCAGAGAGGAGGTTGACTTT
Rela	GTGTTGATAGCTCCTGCTTC	GCAGGGATGTCTCAGACTAA
Nfkb1	GAGGCTACAACTCTGCAAAC	CTCTGTGTAGCCCATCTGTT

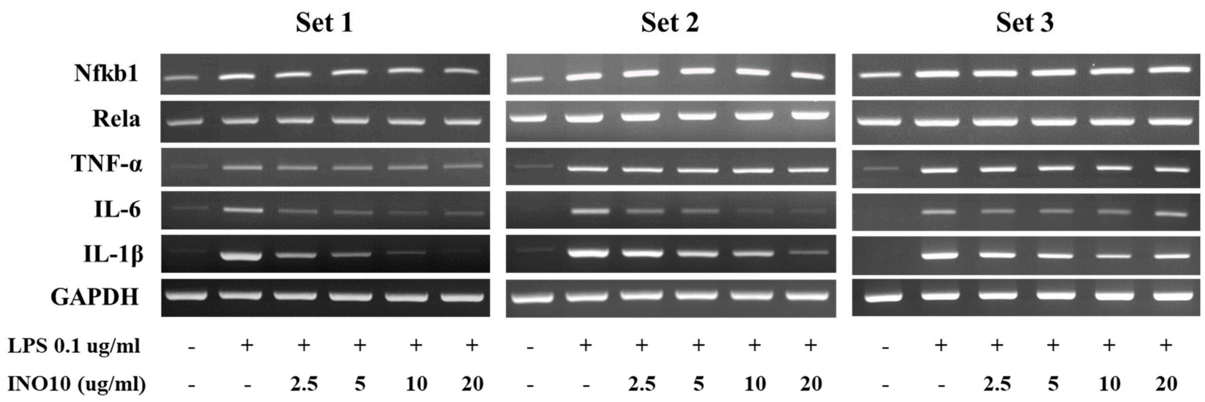

Figure S1. Effects of INO10 on mRNA expressions of Nfkb1, Rela, TNF-α, IL-6 and IL-1β in BV2 cells.

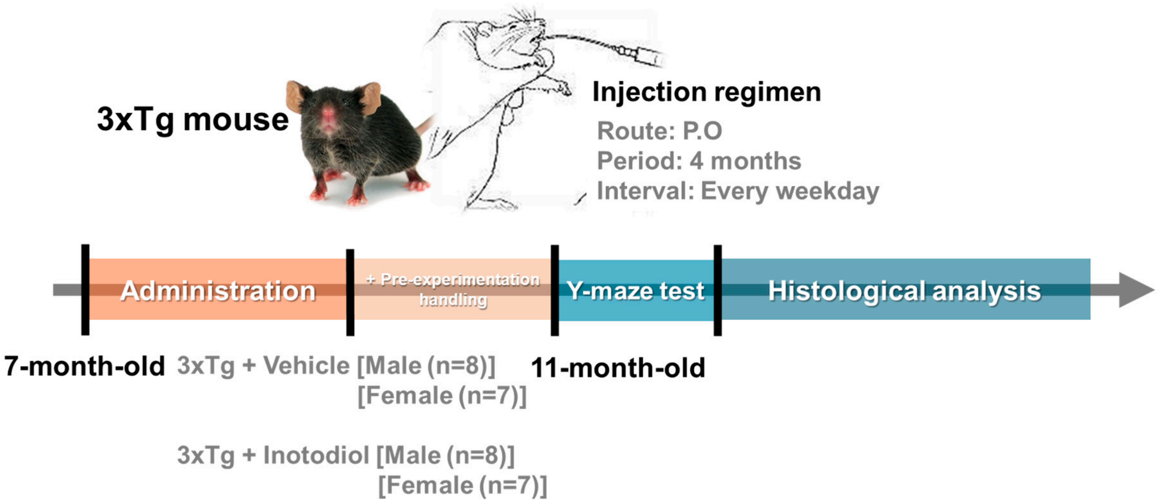

Figure S2. Schematic diagram of the experimental procedure.

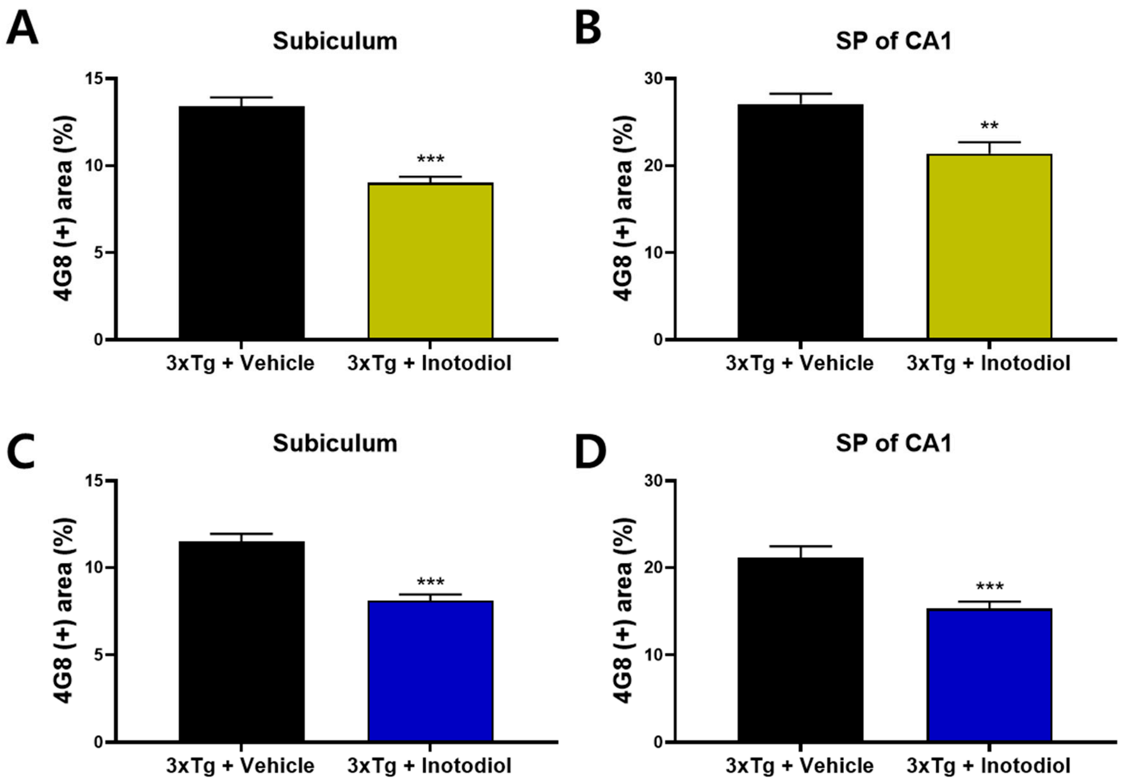

Figure S3. Effect of INO10 on amyloid plaque accumulation in the brain of 11-month-old male and female 3xTg mice. (A, B) The quantitative values of the 4G8-positive area (%) are significantly decreased in the subiculum and CA1 of INO10-administered female 3xTg mice (n=7) compared with the vehicle-administered female 3xTg mice (n=7). (C, D) The quantitative values of the 4G8-positive area (%) are significantly decreased in the subiculum and CA1 of INO10-administered male 3xTg mice (n=8) compared with the vehicle-administered male 3xTg mice (n=8). Data are presented as mean ± SEM. $^{**}p < 0.01$ and $^{***}p < 0.001$ indicate significant differences compared with the vehicle-treated 3xTg mice (black bar).

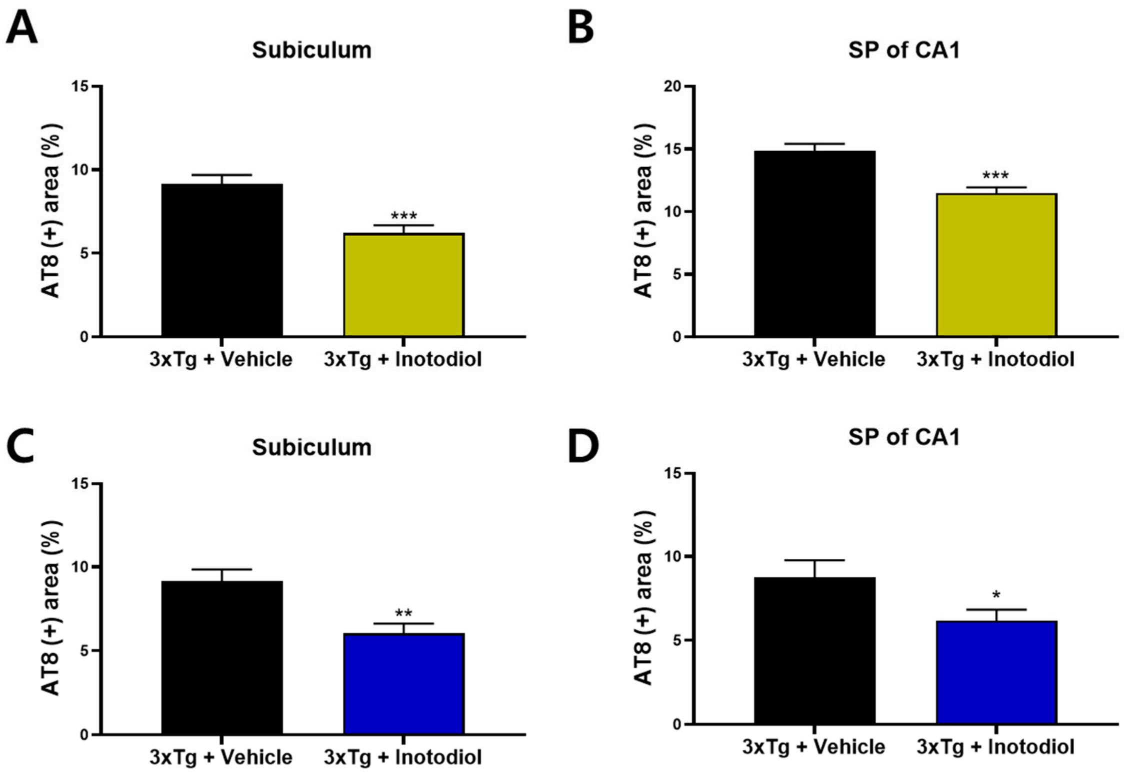

Figure S4. Effect of INO10 against tau phosphorylation at Ser202/Thr205 in the brain of 11-month-old male and female 3xTg mice. (A, B) The quantitative values of the AT8-positive area (%) are significantly decreased in the subiculum and CA1 of INO10-administered female 3xTg mice (n=7) compared with the vehicle-administered female 3xTg mice (n=7). (C, D) The quantitative values of the AT8-positive area (%) are significantly decreased in the subiculum and CA1 of INO10-administered male 3xTg mice (n=8) compared with the vehicle-administered male 3xTg mice (n=8). Data are presented as mean ± SEM. $^{*}p < 0.05$, $^{**}p < 0.01$, and $^{***}p < 0.001$ indicate significant differences compared with the vehicle-treated 3xTg mice (black bar).

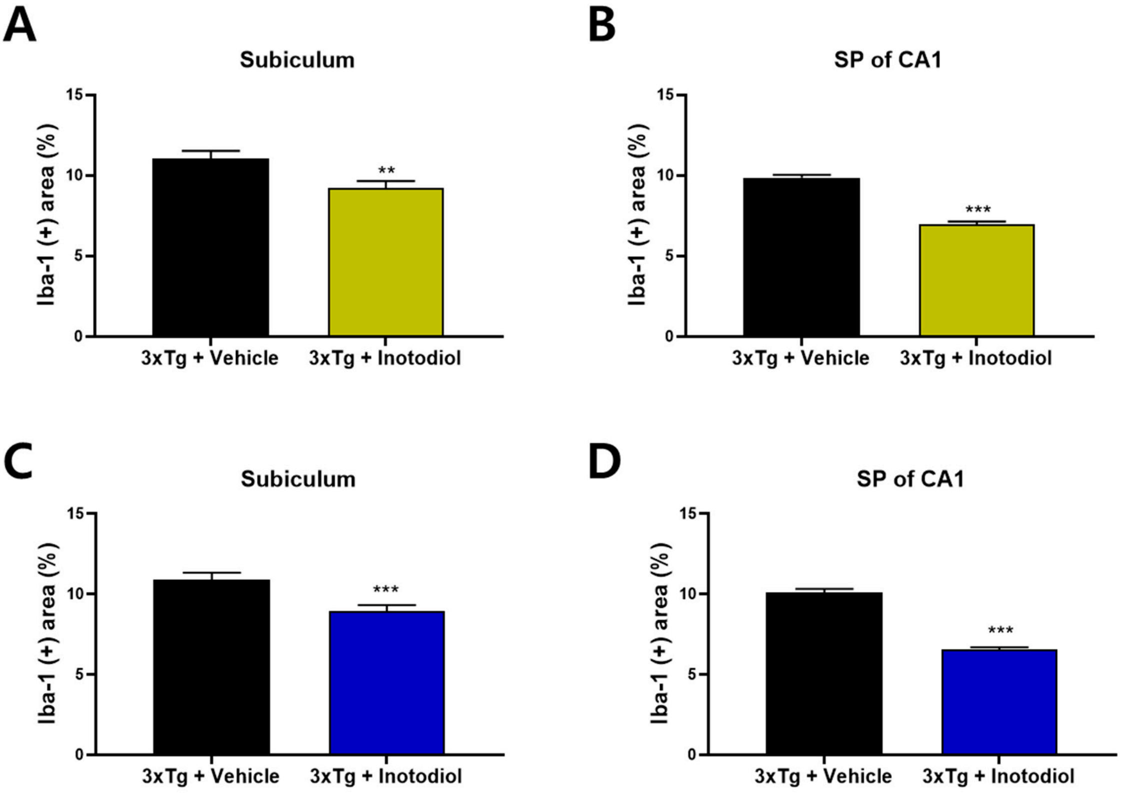

Figure S5. Attenuating effect of INO10 against microgliosis in the subiculum and CA1 of 11-month-old male and female 3xTg mice. (A, B) Quantitative values of the Iba-1-positive area (%) are significantly decreased in the subiculum and CA1 of INO10-administered female 3xTg mice (n=7) compared with the vehicle-administered female 3xTg mice (n=7). (C, D) Quantitative values of the Iba-1-positive area (%) are significantly decreased in the subiculum and CA1 of INO10-administered male 3xTg mice (n=8) compared with the vehicle-administered male 3xTg mice (n=8). Data are presented as mean ± SEM. $^{**}p < 0.01$ and $^{***}p < 0.001$ indicate significant differences compared with the vehicle-treated 3xTg mice (black bar).
